# Bis[1-(3,5-di-*tert*-butyl-2-hydroxy­benz­yl)-3-isopropyl­imidazolium] penta­chlorido(tetra­hydro­furan)samarate(III)–tetrahydrofuran–toluene (1/1/1)

**DOI:** 10.1107/S1600536810017678

**Published:** 2010-05-22

**Authors:** Zhiguo Wang, Siman Liu, Qinquan Bian

**Affiliations:** a Department of Chemistry and Chemical Engineering, Mianyang Normal University, Mianyang 621000, People’s Republic of China

## Abstract

The title compound, (C_21_H_33_N_2_O)_2_[SmCl_5_(C_4_H_8_O)]·C_7_H_8_·C_4_H_8_O, has a layered structure in which each distorted octa­hedral [SmCl_5_(THF)]^2−^ unit (THF is tetra­hydro­furan) is capped by two cations. The central metal Sm^III^ atom of the [SmCl_5_(THF)]^2−^ anionic unit is coordinated by five Cl atoms and one THF O atom, forming a distorted octa­hedral geometry. The crystal structure displays C—H⋯Cl and O—H⋯Cl hydrogen bonding. The structure exhibits disorder of the solvent.

## Related literature

For general background to the use of anionic functionalized *N*-heterocyclic carbenes (NHCs) as anionic tethers in the field of rare earth metals, see: Arnold & Casely (2009 ▶); Arnold & Liddle (2005 ▶, 2006 ▶); Babai & Mudring (2005 ▶); Liddle *et al.* (2007 ▶). For related structures, see: Lu *et al.* (2001 ▶); Wang *et al.* (2006*a* ▶,*b* ▶); Yao *et al.* (2004 ▶, 2007 ▶); Li *et al.* (2005 ▶). 
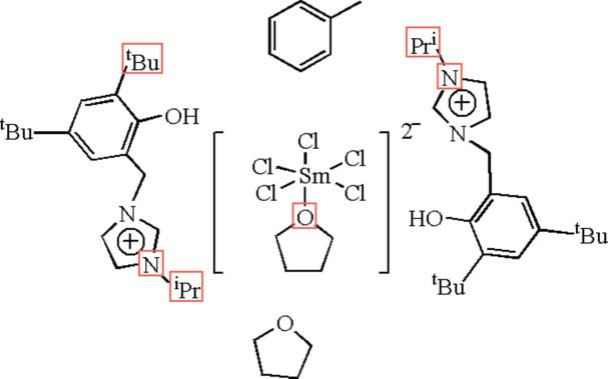


## Experimental

### 

#### Crystal data

(C_21_H_33_N_2_O)_2_[SmCl_5_(C_4_H_8_O)]·C_7_H_8_·C_4_H_8_O
*M* *_r_* = 1222.93
Triclinic, 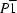

*a* = 10.2252 (11) Å
*b* = 17.6968 (18) Å
*c* = 18.3806 (19) Å
α = 76.954 (5)°
β = 86.077 (6)°
γ = 82.789 (6)°
*V* = 3211.8 (6) Å^3^
*Z* = 2
Mo *K*α radiation
μ = 1.16 mm^−1^
*T* = 192 K
0.34 × 0.25 × 0.15 mm

#### Data collection

Rigaku Mercury diffractometer
Absorption correction: multi-scan (REQAB; Jacobson, 1998 ▶) *T*_min_ = 0.695, *T*_max_ = 0.846
32162 measured reflections
11687 independent reflections
10327 reflections with *I* > 2σ(*I*)
*R*_int_ = 0.031

#### Refinement

*R*[*F* ^2^ > 2σ(*F* ^2^)] = 0.035
*wR*(*F* ^2^) = 0.099
*S* = 1.09
11687 reflections
550 parameters
54 restraints
H-atom parameters constrained
Δρ_max_ = 1.03 e Å^−3^
Δρ_min_ = −0.52 e Å^−3^

### 

Data collection: *CrystalClear* (Rigaku, 2000 ▶); cell refinement: *CrystalClear*; data reduction: *CrystalStructure* (Rigaku/MSC, 2002 ▶); program(s) used to solve structure: *SHELXS97* (Sheldrick, 2008 ▶); program(s) used to refine structure: *SHELXL97* (Sheldrick, 2008 ▶); molecular graphics: *SHELXTL* (Sheldrick, 2008 ▶); software used to prepare material for publication: *SHELXL97*.

## Supplementary Material

Crystal structure: contains datablocks I, global. DOI: 10.1107/S1600536810017678/vm2015sup1.cif

Structure factors: contains datablocks I. DOI: 10.1107/S1600536810017678/vm2015Isup2.hkl

Additional supplementary materials:  crystallographic information; 3D view; checkCIF report

## Figures and Tables

**Table 1:** Hydrogen-bond geometry (Å, °)

*D*—H⋯*A*	*D*—H	H⋯*A*	*D*⋯*A*	*D*—H⋯*A*
O1—H1⋯Cl1	0.84	2.30	3.143 (3)	178
O2—H2⋯Cl3	0.84	2.30	3.136 (3)	174
C29—H29⋯Cl5^i^	0.95	2.54	3.486 (3)	173
C10—H10⋯Cl1^ii^	0.95	2.81	3.681 (4)	153
C9—H9⋯Cl2^iii^	0.95	2.64	3.476 (4)	146
C8—H8⋯Cl5	0.95	2.73	3.655 (4)	164
C5—H5⋯Cl2^iii^	0.95	2.84	3.736 (3)	158

Symmetry codes: (i) 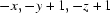; (ii) 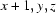; (iii) 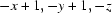.

**Fig. 1.**
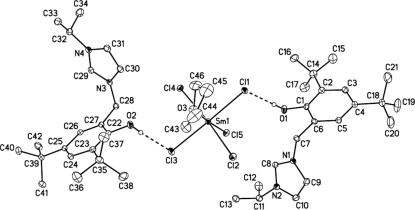
The molecular structure of the title compound showing 30% probability displacement ellipsoids and atom-numbering scheme.

## supplementary crystallographic information

### Comment

Recently, the anionic functionalized N-heterocyclic carbenes( NHCs), such as amino- or phenoxo(alkoxo)-NHCs, have attracted much more attention particularly in the field of rare earth metals as the anionic tether can be used as anchor to enhance the bond of NHC to these electropositive metals (Arnold *et al.*,2006; Liddle *et al.*, 2007). A series of anionic functionalized NHCs have been synthesized and structurally characterized (Arnold *et al.*, 2009; Wang *et al.*, 2006a ). However, the reaction of ligands with cerium triiodide (Arnold *et al.*,2005) or ytterbium trichloride (Wang *et al.*, 2006a) in the presence of organic base gives no desired product. Especially noteworthy is the cleavage reaction of the ligand that occurs during the reaction process ( Wang *et al.*, 2006b ). For a better understanding of the reason, the complex [NdCl_5_(THF)]^2-^[HO-3,5-di-^t^Bu—C_6_H_2_-2-CH_2_{CH(NCHCHN^i^Pr)]^2+^ (Yao *et al.*, 2007) has been studied. In order to further study these compounds, the title complex has been synthesized. Although the ion-pair structure has recently been reported for LnI_3_ in sulfonium-based ionic liquids (Babai *et al.*,2005), its structural feature is different from that of the title complex. In [SEt_3_]_3_[NdI_6_], SEt_3_ is a triethylsulfonium cation; an octahedron [NdI_6_]^3- ^is capped by triethylsulfonium cations (SEt_3_) via the coordination of Nd^3+^to SEt_3_. The X-ray crystal analysis of the title complex indicates that the bis-[(3,5-ditertbutyl-2-hydroxybenzyl)*N*-isopropylimidazolyl] samarium pentachloride consists of an anion [SmCl_5_(THF)]^2-^and two cations [HO-3,5-di-^t^Bu—C_6_H_2_-2-CH_2_{CH(^i^PrNCHCHN)}]^2+^. The central metal Sm in the anionic unit [SmCl_5_(THF)]^2-^ coordinates to five Cl atoms and one oxygen atom from a solvent THF molecule to form a six-coordinated distorted octahedron geometry, which is similar to the reported complex (Yao *et al.*, 2007). The four chlorines Cl(1), Cl(2), Cl(3) and Cl(4) can be considered to occupy the equatorial positions within the octahedron about the lanthanide center. The O(3) and Cl(5) occupy the two axial sites. The angle O(3)—Sm—Cl(5) is slightly distorted from the ideal value of 180° to 174.99 (8)°. The Sm—Cl average bond lengths of 2.6743Å in the title complex are somewhat shorter than 2.789 (2)Å and 2.827Å found in the complex [(SiMe_3_)^2^NC(NC_6_H_11_)_2_]Sm(µ-Cl)_2_Li(THF)_2_ (Yao *et al.*, 2004). The Sm—O(3) bond of 2.452 (3)Å is comparable to 2.448 Å found in the complex [CH_3_C_5_H_4_]_2_Sm[SC(SPh)NPh](THF)]_2_ (Lu *et al.*, 2001). Especially noteworthy is the difference in the network of intermolecular and intramolecular hydrogen bonds. In the complex reported by Yao *et al.* (2007) the hydrogen bonds only exist between the O—H of phenol and Cl. However, in the title complex the hydrogen bonds not only exist in between the O—H of phenol and Cl, but also occur between C—H of imidazole ring, benzyl and isopropyl from the imidazolium ring.

### Experimental

All manipulations were performed under pure argon with rigorous exclusion of air and moisture by using standard Schlenk techniques.THF was degassed and distilled from sodium benzophenone ketyl under argon prior to use. *N*-aryloxo imidazolium chloride [HO-3,5-di-^t^Bu—C_6_H_2_-2-CH_2_{CH(^i^PrNCHCHN)}]Cl was prepared according to the literature (Li *et al.*,2005). A suspension of *N*-aryloxoimidazolium chloride [HO-3,5-di-^t^Bu—C_6_H_2_-2-CH_2_{CH(^i^PrNCHCHN)}]Cl (1.46 g, 4 mol) in THF was added to a suspension of SmCl_3_ (0.52 g, 2 mol) in THF at room temperature. A clean solution was obtained after 10 min under stirring. After concentration and centrifugion, the resulting solution was kept at room temperature in order to obtain colorless crystals (2.05 g, 84% yield). Anal.Calcd. (%) for C_57_H_90_Cl_5_N_4_O_4_Sm: C, 55.93; H, 7.35; N, 4.57; Sm, 12.29. Found: C, 55.76; H, 7.21; N, 4.62; Sm, 12. 32. IR (KBr pellet, cm-1): 3318 (s, v_-OH_); 3125 (s, v_Ar—H_); 3079 (m, v_—C=C—H_); 2958 (s,v_—CH3_); 2868 (m, v_—CH2-_); 1607, 1553, 1482 (m,s, s, v_Ph_); 1457 (s, δ_—CH3_); 1365 (s, δ_—C(CH3)3_); 1224 (s, v_-C—O_); 1149 (s, v_C—N_).

### Refinement

H atoms were positioned geometrically (C—H = 0.95–1.00 A\%) and included in the refinement in the riding-model approximation, with Uiso(H) = 1.2 or 1.5 times Ueq(C). The highest electron-density peak is 0.97 A \% from atom H24. The program SQUEEZE was used to handle the disordered THF and toluene molecules.

### Crystal data

**Table:**

(C_21_H_33_N_2_O)_2_[SmCl_5_(C_4_H_8_O)]·C_7_H_8_·C_4_H_8_O	*Z* = 2
*M**_r_* = 1222.93	*F*(000) = 1098
Triclinic, *P*1	*D*_x_ = 1.264 Mg m^−^^3^
Hall symbol: -P 1	Melting point: 451 K
*a* = 10.2252 (11) Å	Mo *K*α radiation, λ = 0.71070 Å
*b* = 17.6968 (18) Å	Cell parameters from 13058 reflections
*c* = 18.3806 (19) Å	θ = 3.1–25.3°
α = 76.954 (5)°	µ = 1.16 mm^−^^1^
β = 86.077 (6)°	*T* = 192 K
γ = 82.789 (6)°	Block, colourless
*V* = 3211.8 (6) Å^3^	0.34 × 0.25 × 0.15 mm

### Data collection

**Table:**

Rigaku Mercury diffractometer	11687 independent reflections
Radiation source: fine-focus sealed tube	10327 reflections with *I* > 2σ(*I*)
graphite	*R*_int_ = 0.031
Detector resolution: 7.31 pixels mm^-1^	θ_max_ = 25.4°, θ_min_ = 3.1°
ω scans	*h* = −12→12
Absorption correction: multi-scan (REQAB; Jacobson, 1998)	*k* = −20→21
*T*_min_ = 0.695, *T*_max_ = 0.846	*l* = 0→22
32162 measured reflections	

### Refinement

**Table:**

Refinement on *F*^2^	Primary atom site location: structure-invariant direct methods
Least-squares matrix: full	Secondary atom site location: difference Fourier map
*R*[*F*^2^ > 2σ(*F*^2^)] = 0.035	Hydrogen site location: inferred from neighbouring sites
*wR*(*F*^2^) = 0.099	H-atom parameters constrained
*S* = 1.09	*w* = 1/[σ^2^(*F*_o_^2^) + (0.0499*P*)^2^ + 1.7866*P*] where *P* = (*F*_o_^2^ + 2*F*_c_^2^)/3
11687 reflections	(Δ/σ)_max_ = 0.001
550 parameters	Δρ_max_ = 1.03 e Å^−^^3^
54 restraints	Δρ_min_ = −0.51 e Å^−^^3^

### Special details

**Table:**

Geometry. All esds (except the esd in the dihedral angle between two l.s. planes) are estimated using the full covariance matrix. The cell esds are taken into account individually in the estimation of esds in distances, angles and torsion angles; correlations between esds in cell parameters are only used when they are defined by crystal symmetry. An approximate (isotropic) treatment of cell esds is used for estimating esds involving l.s. planes.
Refinement. Refinement of *F*^2^ against ALL reflections. The weighted *R*-factor wR and goodness of fit *S* are based on *F*^2^, conventional *R*-factors *R* are based on *F*, with *F* set to zero for negative *F*^2^. The threshold expression of *F*^2^ > σ(*F*^2^) is used only for calculating *R*-factors(gt) etc. and is not relevant to the choice of reflections for refinement. *R*-factors based on *F*^2^ are statistically about twice as large as those based on *F*, and *R*- factors based on ALL data will be even larger.

### Fractional atomic coordinates and isotropic or equivalent isotropic displacement parameters (Å^2^)

**Table:**

	*x*	*y*	*z*	*U*_iso_*/*U*_eq_	
Sm1	0.175109 (16)	0.492211 (9)	0.245502 (9)	0.02514 (7)	
Cl1	0.09739 (9)	0.41253 (6)	0.14936 (5)	0.0424 (2)	
Cl2	0.36522 (9)	0.54296 (5)	0.14623 (5)	0.0385 (2)	
Cl3	0.23929 (10)	0.58673 (7)	0.32928 (6)	0.0518 (3)	
Cl4	−0.03398 (8)	0.46660 (5)	0.33769 (5)	0.0369 (2)	
Cl5	0.33197 (8)	0.36853 (5)	0.31233 (4)	0.03306 (19)	
O1	0.3259 (3)	0.27589 (16)	0.14977 (13)	0.0406 (6)	
H1	0.2642	0.3122	0.1488	0.061*	
O2	−0.0206 (3)	0.70203 (15)	0.32649 (13)	0.0381 (6)	
H2	0.0504	0.6730	0.3241	0.057*	
O3	0.0360 (3)	0.60325 (15)	0.17351 (16)	0.0510 (7)	
N1	0.5561 (3)	0.38793 (16)	0.07765 (15)	0.0276 (6)	
N2	0.6853 (3)	0.39034 (17)	0.16481 (15)	0.0320 (6)	
N3	−0.2386 (3)	0.61566 (15)	0.45054 (15)	0.0267 (6)	
N4	−0.4509 (3)	0.63699 (16)	0.46579 (15)	0.0294 (6)	
C1	0.3481 (3)	0.2606 (2)	0.07945 (18)	0.0279 (7)	
C2	0.3169 (3)	0.1900 (2)	0.06592 (19)	0.0318 (8)	
C3	0.3536 (4)	0.1754 (2)	−0.0041 (2)	0.0349 (8)	
H3	0.3355	0.1274	−0.0138	0.042*	
C4	0.4156 (3)	0.2270 (2)	−0.06159 (19)	0.0313 (8)	
C5	0.4415 (3)	0.2961 (2)	−0.04605 (18)	0.0297 (7)	
H5	0.4827	0.3328	−0.0836	0.036*	
C6	0.4082 (3)	0.31323 (19)	0.02373 (18)	0.0280 (7)	
C7	0.4343 (3)	0.39133 (19)	0.03719 (19)	0.0302 (7)	
H7A	0.4408	0.4286	−0.0116	0.036*	
H7B	0.3583	0.4117	0.0662	0.036*	
C8	0.5621 (3)	0.3839 (2)	0.14991 (19)	0.0304 (7)	
H8	0.4902	0.3775	0.1855	0.036*	
C9	0.6802 (3)	0.3967 (2)	0.0447 (2)	0.0385 (9)	
H9	0.7045	0.4008	−0.0067	0.046*	
C10	0.7603 (4)	0.3983 (2)	0.0993 (2)	0.0403 (9)	
H10	0.8518	0.4039	0.0935	0.048*	
C11	0.7314 (4)	0.3975 (2)	0.2377 (2)	0.0406 (9)	
H11	0.8274	0.4045	0.2315	0.049*	
C12	0.7150 (5)	0.3238 (3)	0.2962 (2)	0.0550 (11)	
H12A	0.6208	0.3189	0.3068	0.083*	
H12B	0.7562	0.3262	0.3420	0.083*	
H12C	0.7572	0.2786	0.2776	0.083*	
C13	0.6572 (4)	0.4694 (3)	0.2600 (2)	0.0473 (10)	
H13A	0.6666	0.5153	0.2196	0.071*	
H13B	0.6935	0.4769	0.3055	0.071*	
H13C	0.5635	0.4622	0.2694	0.071*	
C14	0.2428 (4)	0.1327 (2)	0.1249 (2)	0.0459 (10)	
C15	0.2191 (6)	0.0613 (3)	0.0949 (3)	0.0696 (15)	
H15A	0.1742	0.0249	0.1340	0.104*	
H15B	0.3039	0.0352	0.0804	0.104*	
H15C	0.1640	0.0783	0.0513	0.104*	
C16	0.1067 (4)	0.1733 (3)	0.1435 (2)	0.0540 (11)	
H16A	0.0589	0.1366	0.1807	0.081*	
H16B	0.0564	0.1904	0.0981	0.081*	
H16C	0.1180	0.2187	0.1637	0.081*	
C17	0.3211 (5)	0.1035 (3)	0.1955 (3)	0.0654 (13)	
H17A	0.3365	0.1480	0.2160	0.098*	
H17B	0.4059	0.0758	0.1832	0.098*	
H17C	0.2711	0.0679	0.2325	0.098*	
C18	0.4527 (4)	0.2058 (2)	−0.1367 (2)	0.0413 (9)	
C19	0.5337 (6)	0.1263 (3)	−0.1269 (3)	0.0852 (18)	
H19A	0.6121	0.1260	−0.0990	0.128*	
H19B	0.5610	0.1157	−0.1762	0.128*	
H19C	0.4803	0.0860	−0.0994	0.128*	
C20	0.5299 (6)	0.2648 (3)	−0.1893 (3)	0.0758 (16)	
H20A	0.4762	0.3156	−0.1999	0.114*	
H20B	0.5525	0.2473	−0.2360	0.114*	
H20C	0.6109	0.2695	−0.1659	0.114*	
C21	0.3270 (6)	0.2028 (5)	−0.1756 (3)	0.112 (3)	
H21A	0.2774	0.1619	−0.1454	0.168*	
H21B	0.3502	0.1910	−0.2248	0.168*	
H21C	0.2727	0.2533	−0.1817	0.168*	
C22	−0.0222 (3)	0.73476 (19)	0.38847 (18)	0.0272 (7)	
C23	0.0019 (3)	0.81275 (19)	0.37987 (19)	0.0300 (7)	
C24	−0.0044 (3)	0.84102 (19)	0.44508 (19)	0.0299 (7)	
H24	0.0119	0.8936	0.4406	0.036*	
C25	−0.0329 (3)	0.79773 (18)	0.51609 (19)	0.0265 (7)	
C26	−0.0586 (3)	0.72138 (18)	0.52153 (18)	0.0252 (7)	
H26	−0.0787	0.6900	0.5692	0.030*	
C27	−0.0556 (3)	0.69004 (18)	0.45866 (19)	0.0271 (7)	
C28	−0.0970 (3)	0.60947 (19)	0.46620 (19)	0.0287 (7)	
H28A	−0.0434	0.5825	0.4307	0.034*	
H28B	−0.0816	0.5783	0.5175	0.034*	
C29	−0.3369 (3)	0.62546 (18)	0.49964 (18)	0.0283 (7)	
H29	−0.3274	0.6244	0.5509	0.034*	
C30	−0.2911 (4)	0.6212 (2)	0.38242 (19)	0.0326 (8)	
H30	−0.2430	0.6171	0.3371	0.039*	
C31	−0.4243 (4)	0.6337 (2)	0.39176 (19)	0.0342 (8)	
H31	−0.4871	0.6391	0.3546	0.041*	
C32	−0.5827 (3)	0.6498 (2)	0.5040 (2)	0.0381 (8)	
H32	−0.5691	0.6650	0.5519	0.046*	
C33	−0.6680 (4)	0.7156 (2)	0.4566 (3)	0.0474 (10)	
H33A	−0.6222	0.7625	0.4445	0.071*	
H33B	−0.7514	0.7261	0.4841	0.071*	
H33C	−0.6861	0.7009	0.4102	0.071*	
C34	−0.6456 (4)	0.5744 (2)	0.5227 (2)	0.0506 (10)	
H34A	−0.6573	0.5576	0.4765	0.076*	
H34B	−0.7317	0.5828	0.5483	0.076*	
H34C	−0.5886	0.5341	0.5553	0.076*	
C35	0.0346 (4)	0.8641 (2)	0.3029 (2)	0.0384 (9)	
C36	0.0482 (5)	0.9481 (2)	0.3087 (2)	0.0618 (13)	
H36A	−0.0328	0.9697	0.3319	0.093*	
H36B	0.0629	0.9802	0.2586	0.093*	
H36C	0.1232	0.9478	0.3393	0.093*	
C37	−0.0761 (5)	0.8674 (3)	0.2496 (2)	0.0575 (12)	
H37A	−0.0823	0.8149	0.2422	0.086*	
H37B	−0.0567	0.9020	0.2015	0.086*	
H37C	−0.1600	0.8875	0.2712	0.086*	
C38	0.1659 (4)	0.8317 (3)	0.2707 (2)	0.0561 (11)	
H38A	0.2371	0.8342	0.3029	0.084*	
H38B	0.1829	0.8628	0.2204	0.084*	
H38C	0.1620	0.7773	0.2682	0.084*	
C39	−0.0354 (3)	0.8352 (2)	0.5834 (2)	0.0322 (8)	
C40	−0.1380 (4)	0.9075 (2)	0.5728 (3)	0.0497 (11)	
H40A	−0.1147	0.9451	0.5273	0.075*	
H40B	−0.1395	0.9316	0.6159	0.075*	
H40C	−0.2253	0.8921	0.5683	0.075*	
C41	0.1010 (4)	0.8604 (2)	0.5890 (2)	0.0409 (9)	
H41A	0.1676	0.8148	0.5944	0.061*	
H41B	0.1006	0.8838	0.6325	0.061*	
H41C	0.1219	0.8989	0.5436	0.061*	
C42	−0.0708 (5)	0.7789 (2)	0.6562 (2)	0.0496 (11)	
H42A	−0.1592	0.7640	0.6536	0.074*	
H42B	−0.0695	0.8045	0.6982	0.074*	
H42C	−0.0065	0.7321	0.6637	0.074*	
C43	0.0734 (5)	0.6803 (3)	0.1423 (3)	0.0660 (13)	
H43A	0.1706	0.6785	0.1360	0.079*	
H43B	0.0413	0.7161	0.1757	0.079*	
C44	0.0120 (7)	0.7071 (4)	0.0691 (3)	0.106 (2)	
H44A	−0.0447	0.7572	0.0670	0.128*	
H44B	0.0805	0.7147	0.0282	0.128*	
C45	−0.0673 (7)	0.6455 (5)	0.0621 (4)	0.112 (2)	
H45A	−0.0209	0.6125	0.0294	0.135*	
H45B	−0.1539	0.6683	0.0413	0.135*	
C46	−0.0841 (5)	0.5992 (3)	0.1404 (4)	0.0826 (17)	
H46A	−0.1608	0.6225	0.1669	0.099*	
H46B	−0.0964	0.5445	0.1408	0.099*	

### Atomic displacement parameters (Å^2^)

**Table:**

	*U*^11^	*U*^22^	*U*^33^	*U*^12^	*U*^13^	*U*^23^
Sm1	0.02691 (10)	0.02751 (10)	0.02159 (10)	−0.00187 (7)	−0.00170 (7)	−0.00708 (7)
Cl1	0.0316 (4)	0.0573 (6)	0.0482 (5)	0.0004 (4)	−0.0091 (4)	−0.0332 (5)
Cl2	0.0455 (5)	0.0389 (5)	0.0300 (5)	−0.0132 (4)	0.0076 (4)	−0.0033 (4)
Cl3	0.0448 (5)	0.0655 (7)	0.0602 (7)	−0.0075 (5)	−0.0042 (5)	−0.0441 (6)
Cl4	0.0286 (4)	0.0490 (5)	0.0344 (5)	−0.0044 (4)	0.0039 (4)	−0.0136 (4)
Cl5	0.0324 (4)	0.0363 (5)	0.0263 (4)	0.0023 (4)	−0.0007 (3)	−0.0018 (3)
O1	0.0477 (16)	0.0485 (16)	0.0253 (13)	0.0031 (12)	−0.0005 (11)	−0.0126 (11)
O2	0.0451 (15)	0.0405 (15)	0.0331 (14)	−0.0058 (12)	0.0042 (11)	−0.0185 (11)
O3	0.0542 (17)	0.0378 (15)	0.0549 (18)	0.0029 (13)	−0.0158 (14)	0.0020 (13)
N1	0.0287 (14)	0.0289 (15)	0.0275 (15)	−0.0038 (12)	−0.0045 (12)	−0.0100 (11)
N2	0.0308 (15)	0.0393 (17)	0.0280 (15)	−0.0036 (13)	−0.0025 (12)	−0.0116 (13)
N3	0.0344 (15)	0.0203 (13)	0.0270 (15)	−0.0060 (12)	−0.0034 (12)	−0.0063 (11)
N4	0.0334 (15)	0.0289 (15)	0.0279 (15)	−0.0066 (12)	−0.0017 (12)	−0.0087 (12)
C1	0.0274 (17)	0.0343 (18)	0.0222 (16)	0.0022 (14)	−0.0018 (13)	−0.0095 (14)
C2	0.0322 (18)	0.0289 (18)	0.0314 (19)	−0.0023 (15)	0.0009 (15)	−0.0015 (14)
C3	0.038 (2)	0.0291 (18)	0.040 (2)	−0.0082 (16)	0.0028 (16)	−0.0121 (15)
C4	0.0285 (17)	0.0353 (19)	0.0329 (19)	−0.0039 (15)	0.0003 (14)	−0.0135 (15)
C5	0.0298 (17)	0.0347 (19)	0.0263 (17)	−0.0071 (15)	0.0009 (14)	−0.0088 (14)
C6	0.0255 (16)	0.0311 (18)	0.0290 (18)	−0.0009 (14)	−0.0073 (14)	−0.0093 (14)
C7	0.0313 (18)	0.0326 (18)	0.0291 (18)	−0.0022 (15)	−0.0091 (14)	−0.0103 (14)
C8	0.0257 (17)	0.0390 (19)	0.0293 (18)	−0.0045 (15)	−0.0016 (14)	−0.0127 (15)
C9	0.0336 (19)	0.055 (2)	0.0280 (19)	−0.0060 (18)	0.0051 (15)	−0.0138 (17)
C10	0.0300 (19)	0.064 (3)	0.0293 (19)	−0.0111 (18)	0.0011 (15)	−0.0120 (18)
C11	0.0314 (19)	0.063 (3)	0.032 (2)	−0.0049 (18)	−0.0089 (16)	−0.0187 (18)
C12	0.068 (3)	0.059 (3)	0.036 (2)	0.006 (2)	−0.018 (2)	−0.009 (2)
C13	0.039 (2)	0.064 (3)	0.049 (2)	−0.006 (2)	−0.0100 (18)	−0.031 (2)
C14	0.057 (3)	0.040 (2)	0.038 (2)	−0.0143 (19)	0.0073 (19)	−0.0015 (17)
C15	0.100 (4)	0.045 (3)	0.064 (3)	−0.034 (3)	0.026 (3)	−0.006 (2)
C16	0.051 (2)	0.068 (3)	0.043 (2)	−0.023 (2)	0.0135 (19)	−0.006 (2)
C17	0.076 (3)	0.059 (3)	0.049 (3)	−0.010 (3)	−0.004 (2)	0.015 (2)
C18	0.047 (2)	0.045 (2)	0.038 (2)	−0.0101 (18)	0.0055 (17)	−0.0205 (17)
C19	0.124 (5)	0.058 (3)	0.069 (3)	0.004 (3)	0.036 (3)	−0.025 (3)
C20	0.114 (5)	0.074 (3)	0.044 (3)	−0.022 (3)	0.023 (3)	−0.025 (2)
C21	0.073 (4)	0.214 (8)	0.076 (4)	−0.016 (5)	−0.003 (3)	−0.093 (5)
C22	0.0245 (16)	0.0288 (17)	0.0296 (18)	−0.0026 (14)	0.0030 (14)	−0.0108 (14)
C23	0.0261 (17)	0.0308 (18)	0.0334 (19)	−0.0058 (14)	0.0005 (14)	−0.0068 (14)
C24	0.0301 (17)	0.0215 (16)	0.039 (2)	−0.0045 (14)	0.0008 (15)	−0.0091 (14)
C25	0.0221 (16)	0.0245 (16)	0.0344 (18)	−0.0013 (13)	−0.0016 (14)	−0.0102 (14)
C26	0.0216 (15)	0.0270 (17)	0.0263 (17)	−0.0015 (13)	−0.0001 (13)	−0.0051 (13)
C27	0.0225 (16)	0.0253 (17)	0.0350 (19)	−0.0004 (13)	−0.0040 (14)	−0.0101 (14)
C28	0.0279 (17)	0.0243 (17)	0.0347 (19)	−0.0017 (14)	−0.0037 (14)	−0.0084 (14)
C29	0.0363 (19)	0.0267 (17)	0.0237 (17)	−0.0036 (14)	−0.0031 (14)	−0.0085 (13)
C30	0.041 (2)	0.0348 (19)	0.0245 (18)	−0.0092 (16)	−0.0002 (15)	−0.0096 (14)
C31	0.041 (2)	0.038 (2)	0.0252 (18)	−0.0090 (16)	−0.0018 (15)	−0.0068 (15)
C32	0.0325 (19)	0.050 (2)	0.036 (2)	−0.0058 (17)	0.0013 (16)	−0.0195 (17)
C33	0.034 (2)	0.036 (2)	0.075 (3)	−0.0008 (17)	−0.004 (2)	−0.021 (2)
C34	0.049 (2)	0.049 (2)	0.050 (3)	−0.012 (2)	0.017 (2)	−0.0042 (19)
C35	0.047 (2)	0.037 (2)	0.0304 (19)	−0.0135 (17)	0.0014 (16)	−0.0028 (15)
C36	0.099 (4)	0.041 (2)	0.044 (2)	−0.026 (3)	0.011 (2)	−0.0002 (19)
C37	0.070 (3)	0.057 (3)	0.042 (2)	−0.008 (2)	−0.011 (2)	0.002 (2)
C38	0.053 (3)	0.070 (3)	0.045 (2)	−0.019 (2)	0.013 (2)	−0.009 (2)
C39	0.0322 (18)	0.0303 (18)	0.038 (2)	−0.0067 (15)	0.0005 (15)	−0.0150 (15)
C40	0.042 (2)	0.047 (2)	0.070 (3)	0.0010 (19)	−0.001 (2)	−0.037 (2)
C41	0.041 (2)	0.044 (2)	0.044 (2)	−0.0092 (18)	−0.0072 (17)	−0.0181 (18)
C42	0.069 (3)	0.054 (3)	0.035 (2)	−0.028 (2)	0.008 (2)	−0.0187 (19)
C43	0.082 (3)	0.037 (2)	0.072 (3)	0.006 (2)	−0.018 (3)	0.000 (2)
C44	0.090 (4)	0.127 (6)	0.066 (4)	0.017 (4)	0.000 (3)	0.038 (4)
C45	0.091 (4)	0.167 (7)	0.075 (4)	0.021 (5)	−0.050 (4)	−0.024 (4)
C46	0.050 (3)	0.069 (3)	0.126 (5)	0.013 (3)	−0.037 (3)	−0.015 (3)

### Geometric parameters (Å, °)

**Table:**

Sm1—O3	2.453 (3)	C20—H20A	0.9800
Sm1—Cl5	2.6546 (9)	C20—H20B	0.9800
Sm1—Cl4	2.6549 (9)	C20—H20C	0.9800
Sm1—Cl2	2.6698 (9)	C21—H21A	0.9800
Sm1—Cl3	2.6789 (9)	C21—H21B	0.9800
Sm1—Cl1	2.7095 (9)	C21—H21C	0.9800
O1—C1	1.377 (4)	C22—C27	1.398 (5)
O1—H1	0.8400	C22—C23	1.404 (5)
O2—C22	1.388 (4)	C23—C24	1.394 (5)
O2—H2	0.8400	C23—C35	1.539 (5)
O3—C46	1.425 (5)	C24—C25	1.388 (5)
O3—C43	1.443 (5)	C24—H24	0.9500
N1—C8	1.319 (4)	C25—C26	1.389 (4)
N1—C9	1.379 (4)	C25—C39	1.526 (4)
N1—C7	1.482 (4)	C26—C27	1.387 (4)
N2—C8	1.331 (4)	C26—H26	0.9500
N2—C10	1.372 (4)	C27—C28	1.512 (4)
N2—C11	1.487 (4)	C28—H28A	0.9900
N3—C29	1.326 (4)	C28—H28B	0.9900
N3—C30	1.374 (4)	C29—H29	0.9500
N3—C28	1.481 (4)	C30—C31	1.358 (5)
N4—C29	1.330 (4)	C30—H30	0.9500
N4—C31	1.382 (4)	C31—H31	0.9500
N4—C32	1.492 (4)	C32—C33	1.506 (5)
C1—C6	1.386 (5)	C32—C34	1.513 (5)
C1—C2	1.406 (5)	C32—H32	1.0000
C2—C3	1.388 (5)	C33—H33A	0.9800
C2—C14	1.537 (5)	C33—H33B	0.9800
C3—C4	1.403 (5)	C33—H33C	0.9800
C3—H3	0.9500	C34—H34A	0.9800
C4—C5	1.377 (5)	C34—H34B	0.9800
C4—C18	1.522 (5)	C34—H34C	0.9800
C5—C6	1.394 (5)	C35—C38	1.528 (6)
C5—H5	0.9500	C35—C37	1.534 (6)
C6—C7	1.516 (4)	C35—C36	1.537 (5)
C7—H7A	0.9900	C36—H36A	0.9800
C7—H7B	0.9900	C36—H36B	0.9800
C8—H8	0.9500	C36—H36C	0.9800
C9—C10	1.344 (5)	C37—H37A	0.9800
C9—H9	0.9500	C37—H37B	0.9800
C10—H10	0.9500	C37—H37C	0.9800
C11—C12	1.510 (6)	C38—H38A	0.9800
C11—C13	1.520 (5)	C38—H38B	0.9800
C11—H11	1.0000	C38—H38C	0.9800
C12—H12A	0.9800	C39—C42	1.529 (5)
C12—H12B	0.9800	C39—C41	1.533 (5)
C12—H12C	0.9800	C39—C40	1.535 (5)
C13—H13A	0.9800	C40—H40A	0.9800
C13—H13B	0.9800	C40—H40B	0.9800
C13—H13C	0.9800	C40—H40C	0.9800
C14—C17	1.526 (6)	C41—H41A	0.9800
C14—C16	1.538 (6)	C41—H41B	0.9800
C14—C15	1.539 (6)	C41—H41C	0.9800
C15—H15A	0.9800	C42—H42A	0.9800
C15—H15B	0.9800	C42—H42B	0.9800
C15—H15C	0.9800	C42—H42C	0.9800
C16—H16A	0.9800	C43—C44	1.478 (7)
C16—H16B	0.9800	C43—H43A	0.9900
C16—H16C	0.9800	C43—H43B	0.9900
C17—H17A	0.9800	C44—C45	1.470 (10)
C17—H17B	0.9800	C44—H44A	0.9900
C17—H17C	0.9800	C44—H44B	0.9900
C18—C20	1.514 (6)	C45—C46	1.496 (9)
C18—C19	1.521 (6)	C45—H45A	0.9900
C18—C21	1.526 (6)	C45—H45B	0.9900
C19—H19A	0.9800	C46—H46A	0.9900
C19—H19B	0.9800	C46—H46B	0.9900
C19—H19C	0.9800		
			
O3—Sm1—Cl5	175.06 (7)	H21A—C21—H21B	109.5
O3—Sm1—Cl4	86.67 (7)	C18—C21—H21C	109.5
Cl5—Sm1—Cl4	96.65 (3)	H21A—C21—H21C	109.5
O3—Sm1—Cl2	84.24 (7)	H21B—C21—H21C	109.5
Cl5—Sm1—Cl2	92.66 (3)	O2—C22—C27	118.7 (3)
Cl4—Sm1—Cl2	170.17 (3)	O2—C22—C23	120.3 (3)
O3—Sm1—Cl3	88.66 (7)	C27—C22—C23	120.9 (3)
Cl5—Sm1—Cl3	95.11 (3)	C24—C23—C22	116.2 (3)
Cl4—Sm1—Cl3	87.62 (3)	C24—C23—C35	121.7 (3)
Cl2—Sm1—Cl3	88.43 (3)	C22—C23—C35	122.0 (3)
O3—Sm1—Cl1	84.52 (7)	C25—C24—C23	124.6 (3)
Cl5—Sm1—Cl1	91.62 (3)	C25—C24—H24	117.7
Cl4—Sm1—Cl1	93.39 (3)	C23—C24—H24	117.7
Cl2—Sm1—Cl1	89.47 (3)	C24—C25—C26	117.0 (3)
Cl3—Sm1—Cl1	173.03 (3)	C24—C25—C39	119.6 (3)
C1—O1—H1	109.5	C26—C25—C39	123.4 (3)
C22—O2—H2	109.5	C27—C26—C25	121.3 (3)
C46—O3—C43	106.3 (3)	C27—C26—H26	119.4
C46—O3—Sm1	125.8 (3)	C25—C26—H26	119.4
C43—O3—Sm1	126.5 (3)	C26—C27—C22	119.9 (3)
C8—N1—C9	108.5 (3)	C26—C27—C28	119.4 (3)
C8—N1—C7	125.8 (3)	C22—C27—C28	120.6 (3)
C9—N1—C7	125.4 (3)	N3—C28—C27	109.9 (3)
C8—N2—C10	108.3 (3)	N3—C28—H28A	109.7
C8—N2—C11	126.2 (3)	C27—C28—H28A	109.7
C10—N2—C11	125.2 (3)	N3—C28—H28B	109.7
C29—N3—C30	108.4 (3)	C27—C28—H28B	109.7
C29—N3—C28	124.4 (3)	H28A—C28—H28B	108.2
C30—N3—C28	126.8 (3)	N3—C29—N4	109.3 (3)
C29—N4—C31	108.3 (3)	N3—C29—H29	125.4
C29—N4—C32	124.2 (3)	N4—C29—H29	125.4
C31—N4—C32	127.6 (3)	C31—C30—N3	107.4 (3)
O1—C1—C6	119.5 (3)	C31—C30—H30	126.3
O1—C1—C2	119.9 (3)	N3—C30—H30	126.3
C6—C1—C2	120.5 (3)	C30—C31—N4	106.7 (3)
C3—C2—C1	116.6 (3)	C30—C31—H31	126.6
C3—C2—C14	121.5 (3)	N4—C31—H31	126.6
C1—C2—C14	121.8 (3)	N4—C32—C33	110.5 (3)
C2—C3—C4	124.3 (3)	N4—C32—C34	109.6 (3)
C2—C3—H3	117.8	C33—C32—C34	112.4 (3)
C4—C3—H3	117.8	N4—C32—H32	108.1
C5—C4—C3	116.8 (3)	C33—C32—H32	108.1
C5—C4—C18	122.6 (3)	C34—C32—H32	108.1
C3—C4—C18	120.6 (3)	C32—C33—H33A	109.5
C4—C5—C6	121.2 (3)	C32—C33—H33B	109.5
C4—C5—H5	119.4	H33A—C33—H33B	109.5
C6—C5—H5	119.4	C32—C33—H33C	109.5
C1—C6—C5	120.5 (3)	H33A—C33—H33C	109.5
C1—C6—C7	120.3 (3)	H33B—C33—H33C	109.5
C5—C6—C7	119.2 (3)	C32—C34—H34A	109.5
N1—C7—C6	113.9 (3)	C32—C34—H34B	109.5
N1—C7—H7A	108.8	H34A—C34—H34B	109.5
C6—C7—H7A	108.8	C32—C34—H34C	109.5
N1—C7—H7B	108.8	H34A—C34—H34C	109.5
C6—C7—H7B	108.8	H34B—C34—H34C	109.5
H7A—C7—H7B	107.7	C38—C35—C37	110.2 (3)
N1—C8—N2	108.9 (3)	C38—C35—C36	107.2 (3)
N1—C8—H8	125.6	C37—C35—C36	108.0 (4)
N2—C8—H8	125.6	C38—C35—C23	110.2 (3)
C10—C9—N1	107.0 (3)	C37—C35—C23	109.8 (3)
C10—C9—H9	126.5	C36—C35—C23	111.4 (3)
N1—C9—H9	126.5	C35—C36—H36A	109.5
C9—C10—N2	107.3 (3)	C35—C36—H36B	109.5
C9—C10—H10	126.3	H36A—C36—H36B	109.5
N2—C10—H10	126.3	C35—C36—H36C	109.5
N2—C11—C12	110.0 (3)	H36A—C36—H36C	109.5
N2—C11—C13	109.6 (3)	H36B—C36—H36C	109.5
C12—C11—C13	112.1 (3)	C35—C37—H37A	109.5
N2—C11—H11	108.3	C35—C37—H37B	109.5
C12—C11—H11	108.3	H37A—C37—H37B	109.5
C13—C11—H11	108.3	C35—C37—H37C	109.5
C11—C12—H12A	109.5	H37A—C37—H37C	109.5
C11—C12—H12B	109.5	H37B—C37—H37C	109.5
H12A—C12—H12B	109.5	C35—C38—H38A	109.5
C11—C12—H12C	109.5	C35—C38—H38B	109.5
H12A—C12—H12C	109.5	H38A—C38—H38B	109.5
H12B—C12—H12C	109.5	C35—C38—H38C	109.5
C11—C13—H13A	109.5	H38A—C38—H38C	109.5
C11—C13—H13B	109.5	H38B—C38—H38C	109.5
H13A—C13—H13B	109.5	C25—C39—C42	111.8 (3)
C11—C13—H13C	109.5	C25—C39—C41	109.0 (3)
H13A—C13—H13C	109.5	C42—C39—C41	109.2 (3)
H13B—C13—H13C	109.5	C25—C39—C40	109.5 (3)
C17—C14—C2	111.0 (3)	C42—C39—C40	108.5 (3)
C17—C14—C16	110.2 (4)	C41—C39—C40	108.9 (3)
C2—C14—C16	109.4 (3)	C39—C40—H40A	109.5
C17—C14—C15	107.9 (4)	C39—C40—H40B	109.5
C2—C14—C15	111.0 (3)	H40A—C40—H40B	109.5
C16—C14—C15	107.2 (4)	C39—C40—H40C	109.5
C14—C15—H15A	109.5	H40A—C40—H40C	109.5
C14—C15—H15B	109.5	H40B—C40—H40C	109.5
H15A—C15—H15B	109.5	C39—C41—H41A	109.5
C14—C15—H15C	109.5	C39—C41—H41B	109.5
H15A—C15—H15C	109.5	H41A—C41—H41B	109.5
H15B—C15—H15C	109.5	C39—C41—H41C	109.5
C14—C16—H16A	109.5	H41A—C41—H41C	109.5
C14—C16—H16B	109.5	H41B—C41—H41C	109.5
H16A—C16—H16B	109.5	C39—C42—H42A	109.5
C14—C16—H16C	109.5	C39—C42—H42B	109.5
H16A—C16—H16C	109.5	H42A—C42—H42B	109.5
H16B—C16—H16C	109.5	C39—C42—H42C	109.5
C14—C17—H17A	109.5	H42A—C42—H42C	109.5
C14—C17—H17B	109.5	H42B—C42—H42C	109.5
H17A—C17—H17B	109.5	O3—C43—C44	106.5 (5)
C14—C17—H17C	109.5	O3—C43—H43A	110.4
H17A—C17—H17C	109.5	C44—C43—H43A	110.4
H17B—C17—H17C	109.5	O3—C43—H43B	110.4
C20—C18—C19	108.1 (4)	C44—C43—H43B	110.4
C20—C18—C4	113.3 (3)	H43A—C43—H43B	108.6
C19—C18—C4	111.2 (3)	C45—C44—C43	106.0 (5)
C20—C18—C21	106.7 (5)	C45—C44—H44A	110.5
C19—C18—C21	108.2 (5)	C43—C44—H44A	110.5
C4—C18—C21	109.1 (3)	C45—C44—H44B	110.5
C18—C19—H19A	109.5	C43—C44—H44B	110.5
C18—C19—H19B	109.5	H44A—C44—H44B	108.7
H19A—C19—H19B	109.5	C44—C45—C46	104.4 (5)
C18—C19—H19C	109.5	C44—C45—H45A	110.9
H19A—C19—H19C	109.5	C46—C45—H45A	110.9
H19B—C19—H19C	109.5	C44—C45—H45B	110.9
C18—C20—H20A	109.5	C46—C45—H45B	110.9
C18—C20—H20B	109.5	H45A—C45—H45B	108.9
H20A—C20—H20B	109.5	O3—C46—C45	103.6 (5)
C18—C20—H20C	109.5	O3—C46—H46A	111.0
H20A—C20—H20C	109.5	C45—C46—H46A	111.0
H20B—C20—H20C	109.5	O3—C46—H46B	111.0
C18—C21—H21A	109.5	C45—C46—H46B	111.0
C18—C21—H21B	109.5	H46A—C46—H46B	109.0
			
Cl4—Sm1—O3—C46	−59.2 (4)	O2—C22—C23—C24	178.7 (3)
Cl2—Sm1—O3—C46	124.6 (4)	C27—C22—C23—C24	2.4 (5)
Cl3—Sm1—O3—C46	−146.9 (4)	O2—C22—C23—C35	−1.7 (5)
Cl1—Sm1—O3—C46	34.5 (4)	C27—C22—C23—C35	−178.0 (3)
Cl4—Sm1—O3—C43	136.1 (3)	C22—C23—C24—C25	−0.2 (5)
Cl2—Sm1—O3—C43	−40.1 (3)	C35—C23—C24—C25	−179.8 (3)
Cl3—Sm1—O3—C43	48.4 (3)	C23—C24—C25—C26	−1.1 (5)
Cl1—Sm1—O3—C43	−130.1 (3)	C23—C24—C25—C39	179.3 (3)
O1—C1—C2—C3	−174.0 (3)	C24—C25—C26—C27	0.2 (5)
C6—C1—C2—C3	2.2 (5)	C39—C25—C26—C27	179.8 (3)
O1—C1—C2—C14	7.5 (5)	C25—C26—C27—C22	2.0 (5)
C6—C1—C2—C14	−176.3 (3)	C25—C26—C27—C28	−174.2 (3)
C1—C2—C3—C4	−1.7 (5)	O2—C22—C27—C26	−179.7 (3)
C14—C2—C3—C4	176.8 (3)	C23—C22—C27—C26	−3.3 (5)
C2—C3—C4—C5	0.4 (5)	O2—C22—C27—C28	−3.6 (5)
C2—C3—C4—C18	179.8 (3)	C23—C22—C27—C28	172.8 (3)
C3—C4—C5—C6	0.5 (5)	C29—N3—C28—C27	−81.4 (4)
C18—C4—C5—C6	−178.9 (3)	C30—N3—C28—C27	90.7 (4)
O1—C1—C6—C5	174.8 (3)	C26—C27—C28—N3	93.6 (3)
C2—C1—C6—C5	−1.5 (5)	C22—C27—C28—N3	−82.5 (4)
O1—C1—C6—C7	−7.4 (5)	C30—N3—C29—N4	0.1 (4)
C2—C1—C6—C7	176.4 (3)	C28—N3—C29—N4	173.5 (3)
C4—C5—C6—C1	0.1 (5)	C31—N4—C29—N3	0.6 (4)
C4—C5—C6—C7	−177.8 (3)	C32—N4—C29—N3	179.7 (3)
C8—N1—C7—C6	−98.4 (4)	C29—N3—C30—C31	−0.8 (4)
C9—N1—C7—C6	88.8 (4)	C28—N3—C30—C31	−173.9 (3)
C1—C6—C7—N1	82.3 (4)	N3—C30—C31—N4	1.1 (4)
C5—C6—C7—N1	−99.8 (4)	C29—N4—C31—C30	−1.0 (4)
C9—N1—C8—N2	0.4 (4)	C32—N4—C31—C30	179.9 (3)
C7—N1—C8—N2	−173.3 (3)	C29—N4—C32—C33	135.9 (3)
C10—N2—C8—N1	−0.3 (4)	C31—N4—C32—C33	−45.2 (5)
C11—N2—C8—N1	172.8 (3)	C29—N4—C32—C34	−99.8 (4)
C8—N1—C9—C10	−0.4 (4)	C31—N4—C32—C34	79.2 (4)
C7—N1—C9—C10	173.4 (3)	C24—C23—C35—C38	115.0 (4)
N1—C9—C10—N2	0.2 (4)	C22—C23—C35—C38	−64.6 (4)
C8—N2—C10—C9	0.1 (4)	C24—C23—C35—C37	−123.5 (4)
C11—N2—C10—C9	−173.1 (3)	C22—C23—C35—C37	57.0 (5)
C8—N2—C11—C12	65.0 (5)	C24—C23—C35—C36	−3.9 (5)
C10—N2—C11—C12	−123.0 (4)	C22—C23—C35—C36	176.6 (3)
C8—N2—C11—C13	−58.7 (5)	C24—C25—C39—C42	179.0 (3)
C10—N2—C11—C13	113.3 (4)	C26—C25—C39—C42	−0.6 (5)
C3—C2—C14—C17	120.4 (4)	C24—C25—C39—C41	−60.2 (4)
C1—C2—C14—C17	−61.2 (5)	C26—C25—C39—C41	120.2 (3)
C3—C2—C14—C16	−117.7 (4)	C24—C25—C39—C40	58.8 (4)
C1—C2—C14—C16	60.7 (5)	C26—C25—C39—C40	−120.9 (3)
C3—C2—C14—C15	0.4 (5)	C46—O3—C43—C44	−25.1 (6)
C1—C2—C14—C15	178.8 (4)	Sm1—O3—C43—C44	142.0 (4)
C5—C4—C18—C20	4.8 (5)	O3—C43—C44—C45	3.5 (7)
C3—C4—C18—C20	−174.6 (4)	C43—C44—C45—C46	18.0 (7)
C5—C4—C18—C19	126.7 (4)	C43—O3—C46—C45	36.1 (6)
C3—C4—C18—C19	−52.7 (5)	Sm1—O3—C46—C45	−131.1 (4)
C5—C4—C18—C21	−113.9 (5)	C44—C45—C46—O3	−33.3 (7)
C3—C4—C18—C21	66.7 (5)		

### Hydrogen-bond geometry (Å, °)

**Table:**

*D*—H···*A*	*D*—H	H···*A*	*D*···*A*	*D*—H···*A*
O1—H1···Cl1	0.84	2.30	3.143 (3)	178
O2—H2···Cl3	0.84	2.30	3.136 (3)	174
C29—H29···Cl5^i^	0.95	2.54	3.486 (3)	173
C10—H10···Cl1^ii^	0.95	2.81	3.681 (4)	153
C9—H9···Cl2^iii^	0.95	2.64	3.476 (4)	146
C8—H8···Cl5	0.95	2.73	3.655 (4)	164
C5—H5···Cl2^iii^	0.95	2.84	3.736 (3)	158

Symmetry codes: (i) −*x*, −*y*+1, −*z*+1; (ii) *x*+1, *y*, *z*; (iii) −*x*+1, −*y*+1, −*z*.
